# Can Sexual Selection Drive the Evolution of Sperm Cell Structure?

**DOI:** 10.3390/cells10051227

**Published:** 2021-05-17

**Authors:** Leigh W. Simmons, Francisco Garcia-Gonzalez

**Affiliations:** 1 Centre for Evolutionary Biology, School of Biological Sciences (M092), The University of Western Australia, Crawley 6009, Australia; paco.garcia@ebd.csic.es; 2 Doñana Biological Station-CSIC, c/Americo Vespucio 26, Isla de la Cartuja, 41092 Sevilla, Spain

**Keywords:** experimental evolution, *Onthophagus*, dung beetles, sperm competition, cryptic female choice, sperm length

## Abstract

Sperm cells have undergone an extraordinarily divergent evolution among metazoan animals. Parker recognized that because female animals frequently mate with more than one male, sexual selection would continue after mating and impose strong selection on sperm cells to maximize fertilization success. Comparative analyses among species have revealed a general relationship between the strength of selection from sperm competition and the length of sperm cells and their constituent parts. However, comparative analyses cannot address causation. Here, we use experimental evolution to ask whether sexual selection can drive the divergence of sperm cell phenotype, using the dung beetle *Onthophagus taurus* as a model. We either relaxed sexual selection by enforcing monogamy or allowed sexual selection to continue for 20 generations before sampling males and measuring the total length of sperm cells and their constituent parts, the acrosome, nucleus, and flagella. We found differences in the length of the sperm cell nucleus but no differences in the length of the acrosome, flagella, or total sperm length. Our data suggest that different sperm cell components may respond independently to sexual selection and contribute to the divergent evolution of these extraordinary cells.

## 1. Introduction

Sperm cells are the most phenotypically diverse cells of metazoan animals [1]. Their evolution has been so divergent that sperm structure alone can be used to construct phylogenetic relationships among species [2,3,4]. Unlike somatic cells, sperm cells must live independently of the diploid parent, in freshwater or marine environments, or within the reproductive tracts of females. They must travel in search of ova with which they must interact and fuse. These stages in the independent life of sperm cells are expected to exert a plethora of selection pressures on performance and so drive their phenotypic evolution. However, our understanding of the evolution of sperm cell diversification remains surprisingly limited.

Rapid and divergent evolution is widely recognized as being a signature of strong directional selection. For example, there is now compelling evidence to show that sexual selection is responsible for the rapid and divergent evolution of secondary sexual traits in males that serve as weapons or ornaments in the competition for access to females [5,6,7,8,9,10]. Arguably, one of the most significant advances in our understanding of sexual selection since Darwin [5], was Geoff Parker’s [11] insight that sexual selection would continue after mating [12]. Because the females of most species will mate with more than one male prior to or during fertilization events, the sperm from multiple males must compete to fertilize a limited supply of ova. Parker [11] recognized that post-mating sexual selection, specifically sperm competition, would favor the evolution of behavioral, physiological, and morphological traits in males that maximize their success in competition for fertilizations. Parker [13] used his insight to argue that sperm competition would favor an evolutionary increase in the numbers of sperm cells males produce, with a consequent reduction in sperm cell size so that sperm competition was responsible for the evolution of anisogamy [12]. Parker’s insight had a radical impact on our evolutionary understanding of reproduction [14]. Evolutionary biologists soon recognized that sperm competition, and the post-mating equivalent of female choice, cryptic female choice [15,16,17], should impose strong selection pressures on sperm cell form and function and likely contribute to their divergent evolution [18,19,20].

There is now considerable evidence from comparative analyses of taxonomic groups, ranging from parasitic worms to mammals, that evolutionary increases in the strength of post-mating sexual selection are associated with increases in male expenditure on sperm production, estimated from relative testes size [21]; the general effect size from meta-analysis of 33 comparative studies using genetic estimates of sperm competition and 66 studies using behavioral estimates of sperm competition are respectively 0.56 and 0.61, with effect size estimates for individual taxa almost always positive [22]. A general effect of sperm competition on sperm numbers is thus widely recognized. However, the effect of sperm competition on sperm cell form and function is less clear. Theoretically, sperm competition is predicted to favor reductions in sperm cell size unless special conditions apply [23]. The findings of comparative analyses of sperm cell length, and the lengths of individual sperm cell components such as the head, mid-piece, and flagellum, have been far more variable than those for relative testes size: positive, negative, and no relationships between measures of sperm cell length and sperm competition have been reported [21]. Overall, however, the general effect size does appear to be positive and significant, though the effect size is smaller than that for testes size and there are clear differences between vertebrate and invertebrate taxa [22]. Effect sizes appear considerably stronger for invertebrates where one of Parker’s special conditions may be satisfied, specifically that sperm competition occurs under high sperm densities such as those expected within the sperm storage organs of female invertebrates [22,24]. Sperm storage organs also facilitate greater interactions between females and sperm cells, and thus greater opportunity for cryptic female choice to affect sperm cell evolution [25]. Indeed, there is good evidence from comparative analyses of a variety of insect taxa that sperm length exhibits correlated evolution with the dimensions of female sperm storage organs or their ducts [26,27,28,29,30,31]. Studies of *Drosophila melanogaster* have demonstrated how interactions between the length of the female seminal receptacle affects the outcome of competitive fertilization events, favoring the extraordinarily long sperm cells found in this genus of fly [32,33,34,35]. Unlike the risk of sperm competition, variation in the strength of cryptic female choice is unlikely to be accurately captured by measures of female remating frequency, be they genetic or behavioral, and sperm competition and cryptic female choice need not favor the same sperm cell traits, making it difficult to draw general conclusions from the findings of comparative analyses.

Despite these issues, the available evidence from comparative analyses does suggest that in general post-mating sexual selection may be associated with the evolution of total sperm length and its component structures [22]. The problem with comparative analyses, however, is that they are correlational and conclusions regarding the causal effect of post-mating sexual selection on sperm cell diversification cannot be drawn. For example, it may be that some other unmeasured variable drives both the evolution of female mating frequency and sperm phenotypes in a similar direction, but that sperm competition and sperm morphology are themselves unrelated. The evidence that sperm cell length, or dimensions of sperm cell components, causally affect sperm performance and competitive fertilization is mixed [21]. While intuitive, sperm length need not in fact translate into faster swimming speeds, and faster swimming sperm might not be optimal for fertilization success in all taxa [21,36]. Thus, our understanding of how sexual selection acts on sperm cell phenotypes is less well developed than our understanding of how sexual selection acts on sperm numbers.

Experimental evolution can offer considerable insight into the causal relationships between selection and phenotypic divergence [37,38]. By manipulating the presence and/or strength of sexual selection acting within and among replicate populations, studies have found divergence among populations in post-mating sexual traits such as testes size, genital morphology, ejaculate quality, and the effects of ejaculates on both male and female fitness [39,40,41,42,43,44,45]. Experimental evolution has also been used to study the effects of sexual selection on sperm cell phenotype, but with mixed results [43,46,47,48]. Here, we manipulate the strength of sexual selection in replicate populations of the bull-horned dung beetle *Onthophagus taurus*, to determine whether sexual selection can affect the microevolutionary divergence of sperm cell phenotypes.

Dung beetles in the genus *Onthophagus* have been used extensively in the testing of sperm competition theory. The beetles arrive at fresh droppings where they dig tunnels in the soil beneath and build a brood ball from dung brought from the surface into which the female lays a single egg [49]. Females mate with multiple males during brood provisioning and store sperm from all of their mating partners in a single sperm storage organ, the spermatheca; on average, sperm competition conforms to a raffle, in which each male sires offspring in proportion to his contribution of sperm to the spermatheca [50,51,52]. Consistent with expectations from sperm competition theory [53], comparative analysis of 16 species of onthophagines revealed a significant evolutionary association between the strength of selection from sperm competition and testes size [54]. A causal relationship between post-mating sexual selection and sperm production has been confirmed using experimental evolution; 20 generations of either enforced monogamy or sexual selection within replicate populations of *O. taurus* found that populations subject to sexual selection evolved larger testes while monogamous populations evolved smaller testes, and divergence in testes size was associated with divergence in competitive fertilization success [55].

Fertilization success in *O. taurus* is also influenced by the length of sperm cells; males with shorter sperm have a fertilization advantage [56]. Here, we quantify variation in sperm length and the lengths of the acrosome, nucleus, and flagella of sperm derived from males following 20 generations of enforced monogamy or sexual selection. We predict that if post-mating sexual selection contributes to the evolution of sperm cell phenotype, then we should see divergence in sperm cell phenotypes among these populations.

## 2. Materials and Methods

### 2.1. Experimental Evolution

Full details of the protocols adopted during experimental evolution have been described elsewhere [55]. In brief, we collected ~1000 beetles from a dairy farm in Byford, Western Australia. From this sample, 300 females were established in breeding tubes, 30 cm in length and 9 cm in diameter, three quarters filled with damp sand and topped with 25 mL of fresh cattle dung. Females were left to construct broods for 1 week, after which tubes were sieved and broods buried in moist sand, in batches of ~50 broods per 10-L box. Broods were incubated at 28 °C for 4 weeks, after which adult offspring had all emerged. Offspring were held in single sex cultures for 1 week to mature sexually. Mixed sex cultures of ~200 beetles were then established in 30-L buckets, containing ~20-L moist sand and topped with 1-L of fresh cattle dung, and left for 1 week to mate. F1 females (300) were recovered from these cultures and established in breeding chambers to produce the next generation. In this way, beetles were bred for two generations to establish them to laboratory conditions.

**Figure 1:**
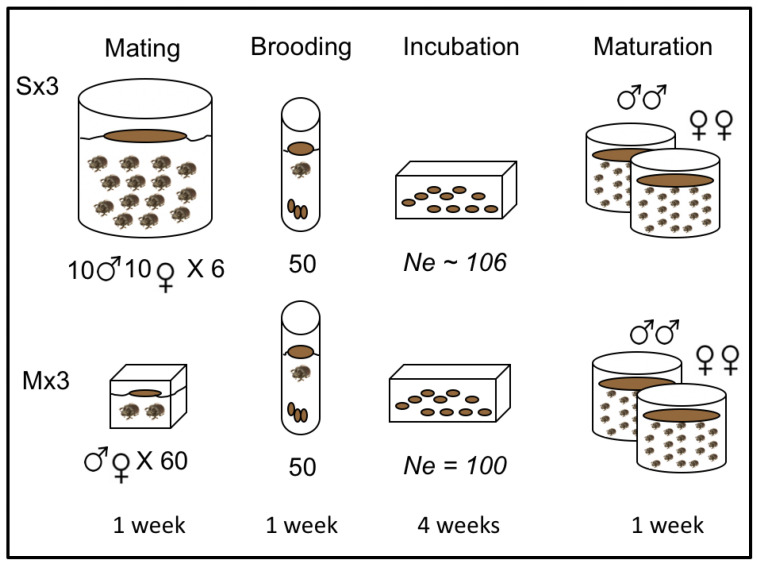
Experimental evolution protocol. S, sexual selection; M, enforced monogamy; *Ne*, effective population size.

Experimental evolution began using the F2 of females collected from the field. We established 3 replicate populations that would evolve under enforced monogamy and 3 replicate populations that would evolve under sexual selection. For each of our monogamous lines, during the mating phase, 60 females were each allocated a single male at random, and the pair housed in a small plastic container (7 × 7 × 5 cm) three-quarters filled with moist sand and topped with 10-mL of fresh dung (Figure 1). For each of our sexual selection lines, 10 males and 10 females were housed in each of 6 30-L buckets for the mating phase of the breeding cycle. All other rearing protocols were identical. Females were established in individual breeding chambers to build broods for 1 week. After sieving broods from individual breeding chambers, we kept broods from the 50 most productive females in each replicate line. These broods were incubated and beetles collected to seed the next generation following the protocols outlined above (Figure 1). The effective population size for monogamous populations was thus 100, and for the sexual selection lines estimated to be in the region of 106, taking into account the average number of different males that females were expected to have mated with and the distribution of paternity among competing males [55]. The standardized Bateman gradient (the relationship between number of mates and male reproductive success) which provides an estimate of the strength of sexual selection acting within the sexual selection populations, was estimated to be ~0.8 [51,55]. Beetles were sampled for this study after 20 generations of experimental evolution.

### 2.2. Sperm Cell Measurement

**Figure 2:**
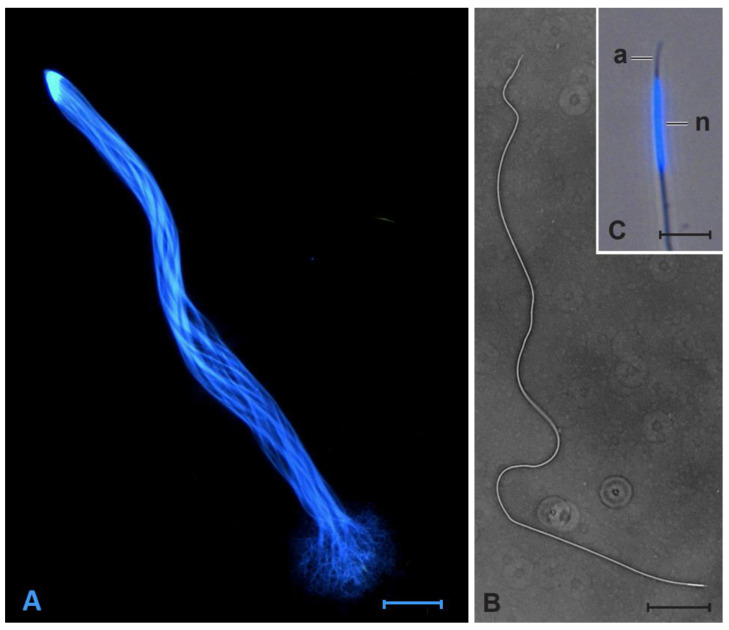
(**A**) A sperm bundle taken from the seminal vesicles of a sexually mature male (scale bar, 100 µm); (**B**) an individual sperm cell (scale bar, 100 µm); (**C**) the head region showing the acrosome (a) and nucleus (n) (scale bar, 10 µm). B and C reprinted by permission from Springer Nature: Springer, Naturwissenschaften, Ultrustructure of spermatozoa of *Onthophagus taurus* (Coleoptera, Scarabaeidae) exhibits heritable variation, Michael Werner and Leigh W. Simmons, ©Springer-Verlag 2011 [57].

A sample of between 16 and 20 (mean ± SE, 18.0 ± 0.6) sexually mature males were collected from each population after they had completed the 1-week of maturation feeding, and frozen at −20 °C. Sperm measurement followed the protocols outlined in Werner and Simmons [57]. Beetles were thawed and dissected. The testicular follicles were separated from the seminal vesicles and discarded. Mature sperm cells were then collected from the seminal vesicles. Mature sperm are joined by their heads to form bundles when in the seminal vesicles (Figure 2), but individual sperm separate from these bundles when transferred to the female (LWS personal observation); individual sperm disassociate from bundles readily when agitated in phosphate buffered saline (PBS). Sperm measurements were made from images captured under light microscopy, using an upright Zeiss AxioImager, equipped with an epifluorescence unit and a Zeiss AxioCam MRc5 digital camera. A 10-μL drop of the sperm suspension in PBS was placed onto a clean glass microscope slide and 2 μL of 4′,6-diamidino-2-phenylindole (DAPI) solution (10 mg/mL) added [57]. After a 10-min incubation period, whole sperm were photographed in phase contrast, and higher magnification images of the head region were taken under fluorescence light (Zeiss Filter Set 01; excitation, BP 365/12; emission, LP 397). The length of the acrosome, the nucleus, and the mitochondrial derivative (hereafter referred to as the flagella, see [57]) of between 9 and 17 (13.6 ± 0.14) sperm cells were measured for each male (Figure 2).

### 2.3. Statistical Analysis

We assessed the within male repeatability of sperm cell components across the sperm cells measured for each male using the R package rptR [58]. We then calculated an average length for each sperm cell component for each individual. We also calculated total sperm length as the sum of each sperm cell component and took the average across all sperm cells sampled for an individual. These average measures of sperm phenotype for each male were used in nested ANOVAs, with treatment as the main effect and replicate population nested within treatment as a random factor. For each male, we also calculated a coefficient of variation in total sperm length and length of each sperm cell component as the standard deviation in each trait divided by its mean and multiplied by 100. Analyses were conducted in JMP v.12.

## 3. Results

**Figure 3:**
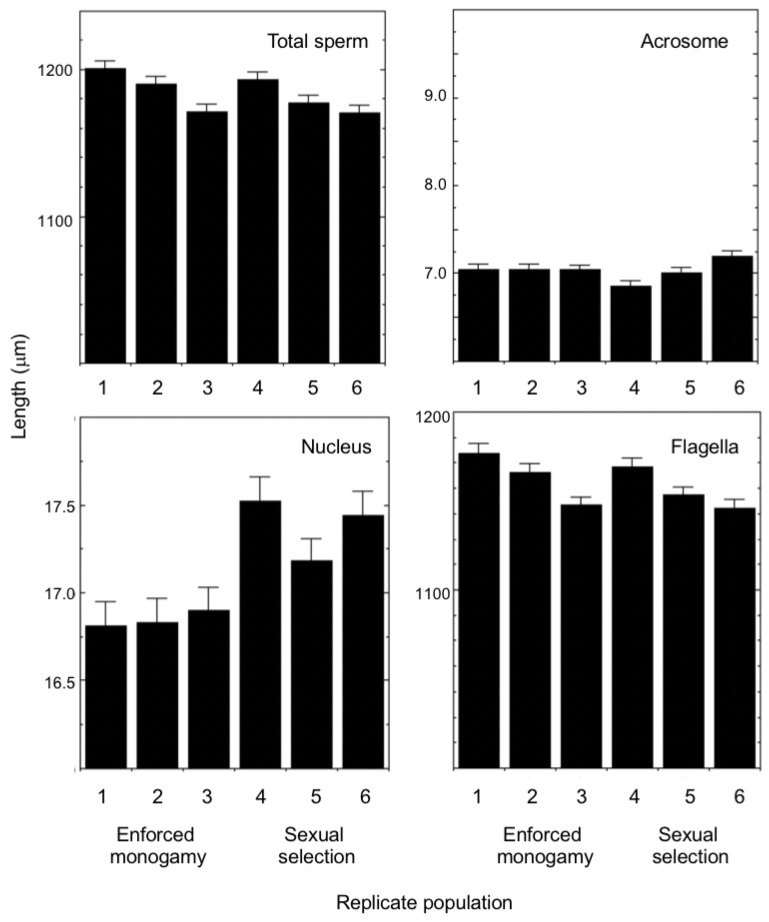
Mean (±SE) total sperm length, and lengths of the acrosome, nucleus, and flagella of males derived from three replicate populations that had been evolving under enforced monogamy (1–3) and three replicate populations that had been evolving under sexual selection (4–6) for 20 generations.

There was significant within-male repeatability for measurements of sperm cell components (R [95% CIs]: acrosome, 0.163[0.155, 0.216], *p* < 0.001; nucleus 0.484 [0.404, 0.552], *p* < 0.001; flagella 0.658 [0.585, 0.715], *p* <0.001). There were no significant correlations between the lengths of constituent parts of the sperm cell (acrosome-nucleus, r = 0.134, *n* = 108, *p* = 0.167; acrosome-flagella, r = 0.109, *n* = 108, *p* = 0.262; nucleus-flagella, r = −0.105, *n* = 108, 0.280). We found no significant divergence in total sperm length, due to our experimental evolution treatments (Table 1). However, analysis of individual sperm cell components revealed that the length of the sperm cell nucleus had diverged in response to sexual selection; males from our sexual selection lines had a longer sperm cell nucleus than males from lines evolving under enforced monogamy (Table 1, Figure 3). The within-male coefficients of variation in total sperm length (1.38 ± 0.04) and the lengths of individual sperm cell components (acrosome: 7.22 ± 0.16; nucleus: 3.57 ± 0.08; flagella: 1.41 ± 0.04) did not vary among our experimentally evolving populations (analyses not shown).

## 4. Discussion

We experimentally manipulated the potential for post-mating sexual selection to act on sperm cell phenotypes in the dung beetle *Onthophagus taurus*. Although we found no significant divergence in the total length of sperm following 20 generations of experimental evolution, we did find significant divergence in the length of the sperm cell nucleus; the sperm cells of males evolving under sexual selection had a longer nucleus than the sperm cells of males evolving under enforced monogamy.

There is evidence to suggest that total sperm length in *O. taurus* is under sexual selection through cryptic female choice. When females mate with two males that differ in sperm length, males with shorter sperm have the greater paternity success [56]. Moreover, a genetic correlation between spermatheca size and sperm length is consistent with a so-called “sexy sperm” process of sperm and spermathecal evolution in this species [59]. All else being equal, the lack of response in total sperm length across generations might therefore seem unexpected.

Evolutionary divergence requires adequate genetic variance, directional selection, and time. Although there is significant additive genetic variance in total sperm length, the mean-standardized coefficient of additive genetic variance (CV_A_) in the field population sampled for this study is considerably lower than that for testes mass, respectively 2.83 compared with 15.59 [60]. Previously, we reported significant divergence in testes mass among these populations [55], suggesting that perhaps the genetic variance in total sperm length was too low to realize a significant divergence. We find this to be unlikely given that we have also found significant divergence in genital traits among these lines, genital traits that have levels of additive genetic variation of a similar magnitude (CV_A_ = 1.69) to sperm length [61].The fact that we found evolutionary divergence in a range of other reproductive traits, including the length of the sperm cell nucleus, also suggests that there was adequate time within which to have seen an evolutionary divergence in total sperm length if it were to happen.

Rather, we suggest that sexual selection on total sperm length may not be directional. While we found that shorter sperm had a selective advantage in fertilization, this effect depended on the size of the female’s spermatheca [56]. Selection on sperm length was only directional when males mated with females with a spermatheca larger than the population average; females with a small spermatheca produced offspring sired by males with intermediate total sperm lengths [56]. Thus, on average, sexual selection across all females may be stabilizing for a total length that matches the population average spermatheca size. Stabilizing sexual selection is predicted to maintain total sperm length across generations, as observed in our experimental evolution. Stabilizing sexual selection should, in theory, generate patterns of slow evolutionary divergence among species. Indeed, comparative analysis across 16 species of onthophagine dung beetles suggest that total sperm length is diverging at a considerably slower rate than either body size or the size of pre-mating secondary sexual traits [9].

In contrast to our findings, Godwin et al. [46] found significant divergence in total sperm length and competitive fertilization success, but not testes mass, following 77 generations of experimental evolution in flour beetles *Tribolium castaneum*; males from male-biased populations had longer sperm than males from female-biased populations [46]. However, consistent with our study, experimental evolution studies that have similarly manipulated the strength of post-mating sexual selection in evolving lines of fruitflies [62], seed beetles [48], and house mice [43] have all reported a lack of divergence in total sperm length. These mixed findings suggest that sexual selection on sperm phenotypes may vary from taxon to taxon and serve to illustrate the complex species-specific relationships that are likely to exist between sperm form and function. Our data suggest that greater understanding will come from studies that have a more nuanced approach to quantifying sperm cell phenotypes than measuring total sperm size.

We found significant divergence in the length of the sperm cell nucleus, suggesting that unlike total sperm length, the nucleus may have been under directional sexual selection in our experimentally evolving lines. We can only speculate on the functional relationship between the length of the sperm cell nucleus and the fertilization processes; little is known for insects in particular [63]. It may be, for example, that the length of the micropile through which the sperm cell must pass to fertilize the ova imposes selection on nucleus length, or that the length of the nucleus affects other aspects of sperm cell movement [64,65], survival in storage, or fertilization competency. Regardless of the functional relationship between nucleus length and fitness in *O. taurus*, our findings support the notion that different components of sperm cells may be subject to different selection pressures and capable of independent evolution. Consistent with this, we found only weak non-significant correlations between sperm cell components. In an experimental evolution approach very similar to the one adopted here, Janick et al. [66] imposed either enforced monogamy or polygamy for 20 generations within independent lines of the flatworm *Macrostomum lignano*. While they found no evolutionary divergence in total sperm cell length, they did find divergence in the length of the lateral bristles [66]. The sperm cell bristles may provide anchorage within the reproductive tract, preventing their removal by the recipient worm following copulation, and so be the target of post-mating sexual conflict [67]. Comparative studies of the rates of evolutionary divergence in components of sperm cells suggest that different components are evolving independently; among lizards and snakes the length of the midpiece appears to be diverging faster than that of the flagella [68,69]. Similarly, among bovids and cervids, the length of the head and the midpiece are diverging faster than the length of the flagella [10]. Among *Drosophila* species that produce two sperm cell types, those that affect fertilization and those that do not, the rate of evolutionary divergence in head length is greater than flagella length for fertile sperm while the reverse is true of the non-fertile sperm morph [70]. These studies all point to the independent evolution of different sperm cell components and demand a more nuanced approach to our study of sperm cell phenotypes. Indeed, it is the ultrastructure of sperm cells that is informative for building phylogenetic relationships among species [2,3,4] and that appears to have undergone particularly rapid and divergent evolution [71]. We suggest that a change in focus from sperm cell size to sperm cell ultrastructure is necessary if we are to understand how post-mating sexual selection drives sperm cell divergence to generate the astonishing variation we see among species in these extraordinary cells.

## Figures and Tables

**Table 1:** Nested analyses of variance in sperm cell components and total sperm length, across 20 generations of three replicate lines of *Onthophagus taurus* subjected to sexual selection and three replicate lines subjected to enforced monogamy. Bonferroni adjusted critical *P*_0.05_ = 0.013 for four related variables.

Measure Effect	MS	df	F	*P*
Total sperm length				
Selection history	1321.28	1	0.41	0.555
Replicate [Selection history]	3197.65	4	6.87	<0.001
Error	47,492.59	102		
Acrosome length				
Selection history	0.02	1	0.06	0.820
Replicate [Selection history]	0.25	4	3.74	0.007
Error	0.07	102		
Nucleus length				
Selection history	7.59	1	25.24	0.007
Replicate [Selection history]	0.30	4	0.90	0.466
Error	0.33	102		
Flagella length				
Selection history	3223.65	1	0.47	0.531
Replicate [Selection history]	3231.33	4	6.96	<0.001
Error	47,351.24	102		

## Data Availability

All data are publicly available via the University of Western Australia Research Repository, https://research-repository.uwa.edu.au/en/datasets/data-from-can-sexual-selection-drive-the-evolution-of-sperm-cell- (accessed on 3 May 2021).
